# Real-World Effectiveness of CARE-Based Spectacle Lenses for Myopia Control in a Turkish Pediatric Cohort

**DOI:** 10.3390/vision10020019

**Published:** 2026-03-31

**Authors:** Nilay Akagun, Ugur Emrah Altiparmak

**Affiliations:** Department of Ophthalmology, Acibadem Hospital Ankara, Yukari Dikmen Tevfik Kis St. No. 6, Ankara 06450, Turkey; emrah.altiparmak@gmail.com

**Keywords:** MyoCare spectacle lenses, myopia control, pediatric myopia, axial length, spherical equivalent refraction, myopia progression, optical myopia control

## Abstract

Childhood myopia progression remains a major global public health concern, and spectacle lenses designed to induce peripheral myopic defocus have emerged as a non-pharmacological strategy for myopia control; however, real-world evidence from European populations remains limited. This retrospective observational study evaluated the 12-month real-world effectiveness of cylindrical annular refractive element spectacle lenses in a Turkish pediatric cohort. Children aged 5–15 years who wore myopia-control spectacle lenses from the CARE platform or single-vision lenses were included. Cycloplegic spherical equivalent refraction (SER) and axial length (AL) were measured at baseline and at 12 months. The primary outcomes were 12-month changes in SER and AL. Multivariable generalized estimation equations were applied to account for inter-eye correlation and to adjust for age and gender. A total of 168 eyes were analyzed (85 with single-vision lenses; 83 with myopia-control lenses). After 12 months, the myopia-control group demonstrated significantly slower progression than the single-vision group, with mean SER changes of −0.40 ± 0.92 D versus −0.77 ± 0.74 D and axial elongation of 0.17 ± 0.25 mm versus 0.31 ± 0.30 mm, respectively. Treatment group remained a significant predictor of both refractive progression (*p* = 0.008) and axial elongation (*p* = 0.003). Age was independently associated with axial length change (*p* < 0.001), whereas gender was not. These findings provide real-world European evidence supporting the role of defocus-modulating spectacle lenses in pediatric myopia management.

## 1. Introduction

Myopia has become one of the most prevalent visual disorders worldwide, affecting an increasing proportion of children and adolescents in both Asian and Western populations [1,2]. The rising incidence of myopia has been closely associated with environmental and behavioral factors such as excessive near work and insufficient outdoor activity [3,4]. Because high myopia substantially increases the risk of irreversible sight-threatening complications—including myopic maculopathy, retinal detachment, and glaucoma—effective strategies to slow its progression have become a global public health priority [5,6].

A variety of optical and pharmacological interventions have been developed to control myopia progression. Among these, low-dose atropine eye drops, orthokeratology lenses, and spectacle lenses designed to induce peripheral myopic defocus are the most widely studied [7,8]. Although atropine is effective, limitations have been reported, such as the need for repeated preparation or renewal, long treatment duration, and light sensitivity, which may reduce long-term adherence in real-world settings [9]. Optical approaches—particularly defocus-based spectacle lens designs—have therefore gained increasing attention due to their non-invasive nature and generally high compliance among pediatric patients [10].

Recent defocus-based optical strategies, such as Defocus Incorporated Multiple Segments (DIMS) and Highly Aspherical Lenslet Target (HALT) technologies, have demonstrated substantial reductions in both refractive progression and axial elongation compared with single-vision lenses [11,12]. Their optical design aims to induce peripheral myopic defocus, which has been proposed as one of the mechanisms contributing to myopia control [13]. Optical modeling studies further suggest that the defocus produced by these designs may differ from theoretical predictions, indicating that additional optical factors—such as peripheral light distribution, retinal contrast modulation, and higher-order aberrations—may also contribute to their treatment effect [14,15]. These findings suggest that the mechanism of action of defocus-based spectacle lenses is likely multifactorial rather than solely attributable to peripheral myopic defocus. This highlights the complexity of treatment effects and suggests that multiple optical and retinal signaling pathways may interact in modulating eye growth.

Cylindrical Annular Refractive Elements (CARE) technology (MyoCare^®^, ZEISS Vision Care, Aalen, Germany) represents a newer optical strategy designed to modulate peripheral defocus through micro-cylindrical annular zones while maintaining central visual clarity. Clinical trials in Asian and European pediatric populations have reported significant reductions in myopia progression and axial elongation compared with single-vision lenses [16,17]. In the present study, spectacle lenses from the CARE platform were evaluated collectively, and analyses were not stratified according to specific CARE lens variants, reflecting real-world prescribing and data availability. However, most available evidence originates from prospective clinical trials conducted under controlled conditions, and real-world data remain limited, particularly in European and Middle Eastern pediatric cohorts [18]. This highlights the need to evaluate the effectiveness of CARE-based spectacle lenses under real-world clinical conditions, where treatment adherence and prescribing patterns may differ from controlled trial settings.

Therefore, the present retrospective study aimed to evaluate the 12-month real-world effectiveness of MyoCare spectacle lenses compared with single-vision lenses (SVLs) in controlling myopia progression and axial elongation in a single-center Turkish pediatric cohort. Changes in spherical equivalent refraction (SER) and axial length (AL) were assessed, and potential influencing factors—including age and gender—were further investigated. This study aims to address the limited availability of real-world evidence on the effectiveness of CARE-based spectacle lenses in non-Asian pediatric populations, where such data remain scarce, thereby underlining the novelty of the present study.

## 2. Materials and Methods

Methods

### 2.1. Ethics

This retrospective comparative observational study was conducted at the Department of Clinical Ophthalmology, Acıbadem Hospital, Ankara, Turkey, in accordance with the tenets of the Declaration of Helsinki. Clinical data obtained during routine care were reviewed. The study protocol was approved by the Acıbadem University Ethics Committee (approval no. 2025-15, decision no. 2025-15/563; 2 October 2025). Due to the retrospective design, the requirement for written informed consent was waived.

### 2.2. Study Period and Compliance

The study included patients who completed 12 months of follow-up between May 2023 and May 2024. All participants were instructed to wear their prescribed spectacles during all waking hours. Compliance with spectacle wear was assessed through repeated interviews with parents and children at follow-up visits. Based on parental reports, the estimated average daily wear time ranged from approximately 12 to 14 h. As compliance was based on parental reporting, recall bias cannot be excluded and may have influenced the interpretation of treatment effectiveness.

### 2.3. Study Population

Children aged 5–15 years who were followed for myopia management were screened for eligibility. Inclusion criteria were as follows: myopic spherical equivalent refraction (SER) ≤ −0.50 diopters in at least one eye; availability of cycloplegic refraction and axial length (AL) measurements at both baseline and 12 months; and continuous wear of either myopia-control spectacle lenses (MyoCare^®^ (ZEISS Vision Care, Aalen, Germany) or single-vision lenses (SVLs) throughout the 12-month follow-up period. The type of spectacle lens prescribed was determined according to routine clinical decision-making and parental preference.

Exclusion criteria included a history of ocular surgery or trauma; amblyopia or strabismus requiring occlusion therapy; keratoconus or other corneal pathology; use of systemic or ocular medications that could affect accommodation; contact lens wear; or incomplete clinical data. When both eyes met the eligibility criteria, data from both eyes were included in the analysis, and inter-eye correlation was addressed using generalized estimation equations.

### 2.4. Cycloplegic Refraction and Axial Length Measurement

Cycloplegic autorefraction was performed using an autorefractometer (Topcon KR-8900; Topcon Corp., Tokyo, Japan) following an instillation of 1% tropicamide ophthalmic solution (Tropamid^®^, Bilim Pharmaceuticals, Istanbul, Turkey). Two drops were administered 5 min apart, and measurements were obtained 30 min after the second instillation. Spherical equivalent refraction (SER) was calculated as the spherical power plus half of the cylindrical power.

This cycloplegia protocol was based on evidence from a randomized clinical trial demonstrating that two instillations of 1% tropicamide provide cycloplegic effects comparable to cyclopentolate in children aged 3–16 years [19]. The mean of three reliable measurements per eye was used to enhance measurement precision, in accordance with established methodological recommendations [20].

Axial length (AL) was measured using an optical biometer (IOLMaster 700; Carl Zeiss Meditec AG, Jena, Germany). The mean of five valid readings per eye was recorded to ensure measurement accuracy. All ocular measurements were obtained by the same experienced examiner under standardized examination conditions. Baseline AL values were categorized according to age- and sex-specific 98th percentile thresholds derived from normative axial length growth curves reported by Tideman et al., which are widely used to define excessive axial elongation in pediatric populations [21].

### 2.5. Outcome Measures

The primary outcomes were the 12-month changes in spherical equivalent refraction (ΔSER) and axial length (ΔAL). Changes were calculated as the difference between baseline and 12-month measurements (follow-up minus baseline).

To evaluate factors associated with myopia progression, multivariable analyses were performed using generalized estimating equations (GEEs) to account for inter-eye correlation resulting from the inclusion of both eyes of each participant. The models included treatment group (single-vision lenses vs. myopia-control spectacle lenses), age, and gender as independent variables.

### 2.6. Statistical Analysis

Statistical analyses were performed using IBM SPSS Statistics (Version 31.0; IBM Corp., Armonk, NY, USA). Continuous variables are presented as mean ± standard deviation, and categorical variables as frequencies and percentages. Baseline comparisons between groups were conducted using the Mann–Whitney U test for continuous variables and the chi-squared test for categorical variables. All analyses were conducted at eye level. Because both eyes of each participant were included in the analyses and were therefore not independent, generalized estimating equations (GEEs) were used to evaluate treatment effects while accounting for inter-eye correlation, in accordance with recommended statistical approaches for ophthalmic data and correlated outcomes [22,23]. An exchangeable working correlation structure was applied. Robust (sandwich) standard errors were used to ensure valid inference despite potential misspecification of the working correlation structure. Separate GEE models were constructed for 12-month spherical equivalent refraction change (ΔSER) and axial length change (ΔAL) as dependent variables. Treatment group and gender were included as fixed factors, and age was entered as a continuous covariate to adjust for its potential influence on myopia progression. All statistical tests were two-sided, and a *p*-value < 0.05 was considered statistically significant.

### 2.7. Post Hoc Power Analysis

A post hoc power analysis was performed using G*Power software (Version 3.1; Heinrich Heine University, Düsseldorf, Germany) [24]. For spherical equivalent refraction, the between-group difference was 0.36 D, corresponding to a moderate effect size (Cohen’s d ≈ 0.55) and an achieved power of approximately 82% (α = 0.05). For axial length, the between-group difference was 0.13 mm, corresponding to a moderate effect size (Cohen’s d ≈ 0.65) and an achieved power of approximately 90% (α = 0.05). These findings indicate that the study was adequately powered to detect clinically meaningful differences.

### 2.8. Data Availability

Statistical code and anonymized data supporting the findings of this study are available from the corresponding author upon reasonable request.

### 2.9. Generative Artificial Intelligence Statement

No generative artificial intelligence (GenAI) tools were used in the design, data collection, analysis, or interpretation of this study.

## 3. Results

### 3.1. Baseline and Demographic Characteristics of the Study Population

Baseline demographic and clinical characteristics are summarized in Table 1A,B. A total of 168 eyes were included in the analysis (SVLs: 85 eyes; CARE-based spectacle lenses: 83 eyes).

Mean age did not differ between groups (SVLs: 10.18 ± 2.32 years; CARE-based spectacle lenses: 10.27 ± 2.80 years; *p* = 0.959). Baseline spherical equivalent refraction was −2.79 ± 1.49 D in the SVL group and −2.92 ± 1.95 D in the CARE-based lens group (*p* = 0.680). Baseline axial length was 24.55 ± 0.94 mm and 24.62 ± 1.12 mm in the SVL and CARE-based lens groups, respectively (*p* = 0.614).

No significant between-group differences were observed in terms of gender distribution (*p* = 0.065) or baseline axial length category distribution (*p* = 0.500).

### 3.2. Twelve-Month Changes and Multivariable GEE Analysis

**Figure 1:**
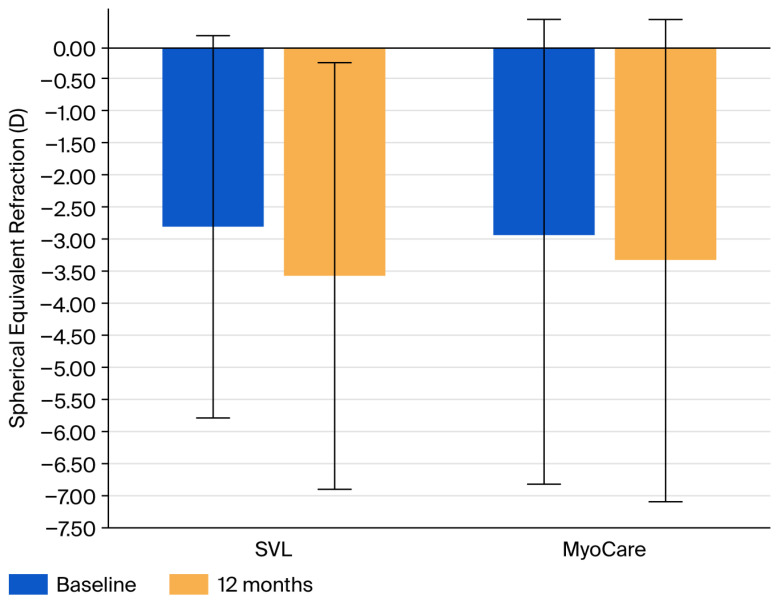
Spherical equivalent refraction (SER) at baseline and 12 months in the MyoCare and single-vision lens (SVL) groups. Error bars represent ±1 standard deviations. Between-group differences at 12 months were statistically significant (*p* < 0.05).

**Figure 2:**
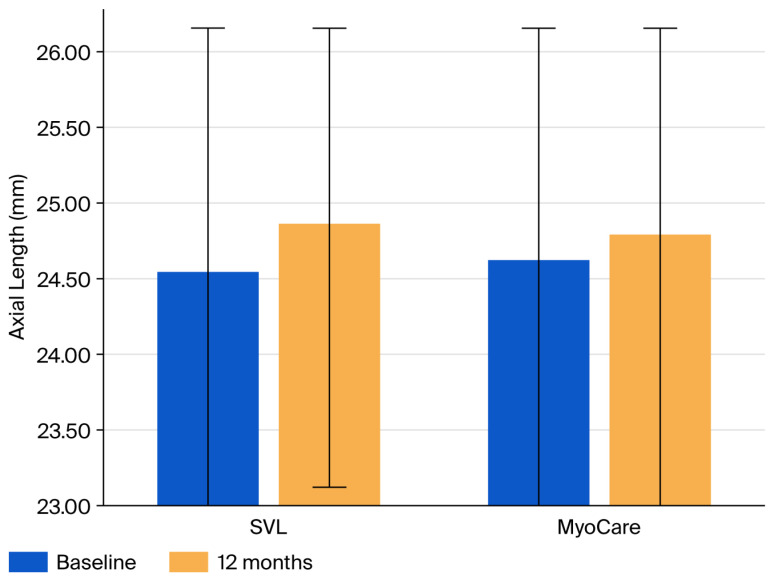
Axial length (AL) at baseline and 12 months in the MyoCare and single-vision lens (SVL) groups. Error bars represent ±1 standard deviations. Between-group differences at 12 months were statistically significant (*p* < 0.05).

At 12 months, both refractive progression and axial elongation were greater in the single-vision lens group than in the MyoCare group, as illustrated in Figure 1 and Figure 2. The mean change in spherical equivalent refraction was −0.77 ± 0.74 D in the SVL group and −0.40 ± 0.92 D in the MyoCare group. Mean axial elongation was 0.31 ± 0.30 mm in the SVL group and 0.17 ± 0.25 mm in the MyoCare group.

In subgroup analyses, boys showed a mean spherical equivalent change of −0.50 ± 0.66 D and girls showed a change of −0.65 ± 0.98 D. Mean axial elongation was 0.23 ± 0.23 mm in boys and 0.26 ± 0.32 mm in girls. Eyes with low–moderate baseline axial length demonstrated a mean refractive change of −0.64 ± 0.78 D and axial elongation of 0.29 ± 0.34 mm, whereas eyes with high baseline axial length showed a mean refractive change of −0.53 ± 0.95 D and axial elongation of 0.19 ± 0.22 mm (Table 2).

Treatment group was significantly associated with both refractive progression and axial elongation in the multivariable GEE models. Eyes in the MyoCare group showed significantly less myopia progression than those in the single-vision lens group (B = −0.360, 95% CI −0.627 to −0.092, *p* = 0.008). Similarly, treatment group remained significantly associated with axial elongation (B = 0.130, 95% CI 0.044 to 0.216, *p* = 0.003).

Age was not significantly associated with spherical equivalent change (*p* = 0.137) but was significantly associated with axial length change (B = −0.025, 95% CI −0.036 to −0.015, *p* < 0.001). Gender, baseline SER, and baseline axial length group were not significantly associated with refractive or axial length changes in the multivariable models (all *p* > 0.05) (Table 3).

## 4. Discussion

The present study provides real-world evidence that myopia-control spectacle lenses were associated with significantly slower myopia progression and axial elongation over 12 months compared with single-vision lenses. Importantly, these associations remained statistically significant after adjustment for potential confounding factors, including age, gender, baseline refractive error, and baseline axial length, using multivariable generalized estimating equation models that accounted for inter-eye correlation. These findings provide further evidence supporting the potential effectiveness of defocus-modulating spectacle lenses in real-world clinical settings.

In the multivariable models, treatment group was significantly associated with both refractive progression and axial elongation. Eyes wearing myopia-control spectacle lenses demonstrated significantly less myopic progression and reduced axial length increase compared with eyes wearing single-vision lenses. These findings suggest that the observed associations were not solely explained by baseline clinical differences between groups. Age was not significantly associated with refractive change but was significantly associated with axial elongation, with increasing age related to smaller axial length increases. This finding is consistent with the known physiological deceleration of ocular growth during later childhood and underscores the importance of accounting for age when evaluating axial elongation in pediatric populations [25].

Our findings are consistent with the results of the Clinical Evaluation of MyoCare in Europe (CEME) trial, the first randomized controlled study evaluating cylindrical annular refractive element spectacle lenses in European children [26]. In the CEME study, CARE lenses reduced myopia progression by 0.21 D and axial elongation by 0.14 mm compared with single-vision lenses at 12 months. In the present real-world cohort, the magnitude of the between-group differences was comparable for axial elongation and somewhat greater for refractive progression, with differences of 0.36 D for spherical equivalent refraction and 0.13 mm for axial length.

Several factors may explain these differences, which may be related to variations in baseline myopia severity, cohort characteristics, and treatment adherence patterns between study populations. Unlike the randomized and tightly controlled design of the CEME trial, the present study reflects real-world prescription and follow-up patterns in routine pediatric ophthalmic practice. Despite these methodological differences, both studies consistently demonstrate that cylindrical annular refractive element spectacle lenses are associated with clinically meaningful reductions in myopia progression and axial elongation in European pediatric populations. Real-world studies may be influenced by variations in patient adherence, prescribing behavior, and follow-up patterns, which can lead to differences in observed treatment effects compared with randomized controlled trials. Therefore, treatment effects observed in real-world cohorts should be interpreted while considering the methodological differences between observational studies and randomized controlled trials. However, given the observational design of the study, residual confounding cannot be excluded and may have influenced the observed associations.

When compared with the findings of Liu et al. [27], who evaluated a cylindrical annular refractive element spectacle lens in children aged 8–12 years, our results demonstrate a somewhat greater reduction in refractive progression and a comparable reduction in axial elongation over 12 months. In the Liu cohort, CARE lenses reduced spherical equivalent progression by 0.15 D (−0.56 D vs. −0.71 D) and axial elongation by 0.08 mm (0.27 mm vs. 0.35 mm) compared with single-vision lenses. In contrast, our study observed between-group differences of 0.36 D for refractive progression and 0.13 mm for axial elongation.

A one-year randomized clinical trial by Chen et al. [28] compared CARE and CARE-S lenses with single-vision lenses in children aged 6–13 years and demonstrated significant reductions in both myopia progression and axial elongation. The comparable efficacy of the two optical designs suggests that defocus-modulating spectacle lenses can achieve clinically meaningful myopia control across different configurations. Consistent with these randomized findings, our real-world cohort also showed significantly reduced refractive progression and axial elongation among children wearing myopia-control spectacle lenses. Together, these results indicate that the effectiveness of defocus-modulating spectacle lenses may extend beyond controlled trial settings into everyday clinical practice.

The same cohort was subsequently followed for two years, providing important evidence on treatment durability. In the 24-month follow-up of the randomized trial by Chen et al. [29], children aged 6–13 years remained randomized irrespective of age to single-vision, CARE, or CARE-S spectacle lenses. Both CARE and CARE-S lenses continued to demonstrate sustained and clinically meaningful myopia-control effects. Spherical equivalent progression reached −1.15 ± 0.63 D in the single-vision group, compared with −0.73 ± 0.63 D and −0.80 ± 0.56 D in the CARE and CARE-S groups, respectively. Axial elongation followed a similar pattern, measuring 0.59 ± 0.26 mm in the single-vision group versus 0.40 ± 0.26 mm and 0.44 ± 0.25 mm in the treatment groups.

Despite the optical design differences between CARE (+4.6 D, 7 mm central clear zone) and CARE-S (+3.8 D, 9 mm central clear zone), no clinically meaningful difference in efficacy was detected between the two lenses, suggesting durable treatment effects across a broad pediatric age range.

Previous studies using different optical myopia-control designs have consistently reported age-dependent treatment responses, with stronger effects observed in younger children. Studies evaluating Defocus Incorporated Multiple Segments (DIMS) lenses and highly aspherical lenslet (HAL) designs have both demonstrated greater treatment efficacy in younger age groups, with progressively reduced effects in older children [30,31,32,33,34]. In the present study, age was not significantly associated with refractive change but was significantly associated with axial length change, with older age being linked to smaller axial elongation. This pattern may reflect the different biological and measurement characteristics of refractive and biometric outcomes [35]. Refractive error is influenced by multiple optical components and may show greater variability, whereas axial length provides a more direct marker of ocular growth [35]. Our findings therefore support the concept that age-related differences in treatment response may be more consistently detected when axial elongation is used as the primary outcome measure.

In addition to age-related effects, the magnitude of the treatment effect observed in the present study can be compared with those in previous studies evaluating defocus-modulating spectacle lenses. In a study by Bao et al. [33], axial elongation in the single-vision group was 0.36 mm, with a reduction of 0.23 mm in the HAL group, and spherical equivalent progression showed a between-group difference of 0.53 D. Similarly, real-world data on DIMS lenses reported a spherical equivalent progression of −0.28 D versus −0.74 D and axial elongation of 0.14 mm versus 0.29 mm.

In comparison, the present study demonstrated an axial elongation of 0.31 mm versus 0.17 mm (difference: 0.13 mm) and a spherical equivalent progression of −0.77 D versus −0.40 D (difference: 0.36 D). Although slightly smaller than those reported in randomized trials, these effects remain comparable and clinically meaningful.

Finally, although several studies evaluating cylindrical annular refractive element spectacle lenses have been published in recent years, the overall evidence base remains limited, particularly for real-world cohorts outside Asia. Most previous investigations have relied on randomized clinical trial designs, whereas real-world evidence reflecting routine clinical prescribing and follow-up remains limited. The present study therefore provides important real-world data from a Turkish pediatric cohort and represents one of the early European evaluations of these spectacle lenses under everyday clinical conditions.

Clinical Implications

These findings may assist clinicians in refining optical prescribing decisions by highlighting the potential effectiveness of myopia-control spectacle lenses in slowing both refractive progression and axial elongation in routine clinical practice. The results also emphasize the importance of considering age-related ocular growth dynamics when interpreting treatment outcomes and monitoring responses in pediatric myopia management. However, these findings should be interpreted with caution given the retrospective design and relatively modest sample size of the study.

Strengths and Limitations

This study has several notable strengths. It provides real-world evidence on the performance and potential effectiveness of cylindrical annular refractive element spectacle lenses in a Turkish pediatric population, contributing to a European clinical setting where evidence remains limited. The inclusion of both eyes, with inter-eye correlation appropriately addressed through generalized estimation equation modeling, strengthens the statistical robustness of the analyses. In addition, standardized cycloplegic refraction and axial length measurement protocols ensured methodological consistency and enhanced the reliability of the biometric and refractive outcomes.

Several limitations should also be acknowledged. The retrospective and non-randomized study design may introduce selection bias and inherently limit causal inference. Compliance with spectacle wearing was based on parental reporting rather than objective monitoring, which may reduce the accuracy of adherence assessment. Lens allocation was determined through routine clinical decision-making and parental preference rather than randomization, reflecting real-world practice but differing from experimental protocols used in randomized controlled trials. Because lens allocation was influenced by routine clinical decision-making and parental preference, socioeconomic factors and other unmeasured variables may have contributed to selection bias and residual confounding. This may have introduced systematic differences between groups, potentially influencing the observed treatment effects.

The relatively modest sample size may limit the generalizability of the findings. Moreover, environmental and behavioral factors—including parental myopia, near-work activities, digital screen exposure, and outdoor time—were not quantified and therefore could not be incorporated into the multivariable analyses, potentially contributing to residual confounding. In addition, as this was a single-center study, local clinical practices and prescribing patterns may have influenced treatment selection and outcomes, which should be considered when interpreting the generalizability of the findings. Although tropicamide was used for cycloplegia in accordance with routine clinical practice, its cycloplegic effect may be weaker than that of cyclopentolate in pediatric populations, which should be considered when interpreting refractive measurements.

## 5. Conclusions

In this real-world pediatric cohort, cylindrical annular refractive element spectacle lenses were associated with significantly slower myopia progression and reduced axial elongation compared with single-vision lenses. Treatment group remained significantly associated with both refractive and axial outcomes after adjustment for age and gender, suggesting the potential effectiveness of these lenses under routine clinical practice conditions.

These findings provide real-world evidence from a European population and reinforce the role of defocus-modulating spectacle lenses as a potentially effective component of contemporary pediatric myopia management.

## Figures and Tables

**Table 1:** Baseline characteristics of the study groups. (A) Continuous variables; (B) categorical variables.

**(A) Continuous Variables**					
**Variable**	**SVL (n = 85)**		**MyoCare (n = 83)**		***p*-Value**
Age (years)	10.18 ± 2.32 (6–15)		10.27 ± 2.80 (5–15)		0.959
SER_1_ (D)	−2.79 ± 1.49 (−0.75–−7.50)		−2.92 ± 1.95 (−0.75–−9.38)		0.68
AL_1_ (mm)	24.55 ± 0.94 (22.51–27.13)		24.62 ± 1.12 (22.14–27.85)		0.614
**(B) Categorical Variables**					
**Variable**	**Category**	**SVL (n = 85)**	**MyoCare (n = 83)**	**Total (n = 168)**	***p*-Value**
Gender	Boys	33 (38.8%)	44 (53.0%)	77 (45.8%)	0.065
	Girls	52 (61.2%)	39 (47.0%)	91 (54.2%)	
Baseline AL	Low–moderate	43 (50.6%)	41 (49.4%)	84 (51.2%)	0.5
	High	42 (49.4%)	42 (50.6%)	84 (48.8%)	

Note: Continuous variables are presented as mean ± standard deviation (SD); ranges are shown as minimum–maximum values. Group comparisons were performed using Mann–Whitney U tests. D = diopters; mm = millimeters; SER = spherical equivalent refraction; AL = axial length. Categorical variables are presented as n (%). Group comparisons were performed using chi-squared tests.

**Table 2:** Twelve-month changes in spherical equivalent refraction and axial length across study subgroups.

Outcome	Subgroup	N (Eyes)	Mean ± SD
SER Change (D)	SVL	85	−0.77 ± 0.74
	MyoCare	83	−0.40 ± 0.92
	Boys	77	−0.50 ± 0.66
	Girls	91	−0.65 ± 0.98
	Low–moderate baseline AL	84	−0.64 ± 0.78
	High baseline AL	80	−0.53 ± 0.95
AL Change (mm)	SVLs	85	0.31 ± 0.30
	MyoCare	83	0.17 ± 0.25
	Boys	77	0.23 ± 0.23
	Girls	91	0.26 ± 0.32
	Low–moderate baseline AL	84	0.29 ± 0.34
	High baseline AL	80	0.19 ± 0.22

Note: Continuous variables are presented as mean ± standard deviation (SD). n indicates the number of eyes. Negative SER change represents myopic progression. Baseline AL groups were defined according to age-adjusted normative percentiles. D = diopters; mm = millimeters; SER = spherical equivalent refraction; AL = axial length.

**Table 3:** Multivariable GEE models identifying predictors of 12-month spherical equivalent and axial length change.

Outcome	Predictor	Β (B)	95% CI	Wald χ^2^	*p*-Value
SER change (D)	Treatment	**−0.360**	−0.627 to −0.092	6.957	**0.008**
	Gender	0.064	−0.156 to 0.285	0.327	0.567
	Baseline AL group	0.040	−0.479 to 0.559	0.023	0.881
	Age (years)	0.027	−0.009 to 0.062	2.206	0.137
	Baseline SER	−0.086	−0.322 to 0.150	0.507	0.476
AL change (mm)	Treatment	**0.130**	0.044 to 0.216	8.833	**0.003**
	Gender	−0.017	−0.107 to 0.072	0.140	0.709
	Baseline AL group	0.0620	−0.025 to 0.148	1.968	0.161
	**Age (years)**	**−0.025**	−0.036 to −0.015	23.142	**<0.001**

Note: Results are derived from multivariable generalized estimating equation (GEE) models accounting for inter-eye correlation. B represents the regression coefficient. Wald χ^2^ tests the significance of each predictor in the model. CI = confidence interval. Negative coefficients indicate slower myopia progression or reduced axial elongation. D = diopters; mm = millimeters; SER = spherical equivalent refraction; AL = axial length.

## Data Availability

The data that support the findings of this study are available from the corresponding author upon reasonable request. The data are not publicly available due to privacy and ethical restrictions.

## Abbreviations

The following abbreviations are used in this manuscript:

AL	Axial Length
SER	Spherical Equivalent Refraction
SVL	Single-Vision Lens
GEEs	Generalized Estimating Equations
SD	Standard Deviation
